# Distinguishing infectivity in patients with pulmonary tuberculosis using deep learning

**DOI:** 10.3389/fpubh.2023.1247141

**Published:** 2023-11-27

**Authors:** Yi Gao, Yiwen Zhang, Chengguang Hu, Pengyuan He, Jian Fu, Feng Lin, Kehui Liu, Xianxian Fu, Rui Liu, Jiarun Sun, Feng Chen, Wei Yang, Yuanping Zhou

**Affiliations:** ^1^Department of Infectious Disease and Hepatology Unit, Nanfang Hospital, Southern Medical University, Guangzhou, China; ^2^Department of Infectious Disease, Hainan General Hospital, Hainan Medical University, Haikou, China; ^3^Department of Gastroenterology, Nanfang Hospital, Southern Medical University, Guangzhou, China; ^4^School of Biomedical Engineering, Southern Medical University, Guangzhou, China; ^5^Department of Infectious Disease, The Fifth Affiliated Hospital, Sun Yat-sen University, Zhuhai, China; ^6^Department of Radiology, Haikou Municipal People's Hospital and Central South University Xiangya Medical College Affiliated Hospital, Haikou, China; ^7^Clinical Lab, Haikou Municipal People's Hospital and Central South University Xiangya Medical College Affiliated Hospital, Haikou, China; ^8^Department of Infectious Disease, The Second Affiliated Hospital, Hainan Medical University, Haikou, China; ^9^Department of Radiology, Hainan General Hospital, Hainan Medical University, Haikou, China

**Keywords:** pulmonary tuberculosis, deep learning, disease control and prevention, infectivity identification, CT

## Abstract

**Introduction:**

This study aimed to develop and assess a deep-learning model based on CT images for distinguishing infectivity in patients with pulmonary tuberculosis (PTB).

**Methods:**

We labeled all 925 patients from four centers with weak and strong infectivity based on multiple sputum smears within a month for our deep-learning model named TBINet's training. We compared TBINet's performance in identifying infectious patients to that of the conventional 3D ResNet model. For model explainability, we used gradient-weighted class activation mapping (Grad-CAM) technology to identify the site of lesion activation in the CT images.

**Results:**

The TBINet model demonstrated superior performance with an area under the curve (AUC) of 0.819 and 0.753 on the validation and external test sets, respectively, compared to existing deep learning methods. Furthermore, using Grad-CAM, we observed that CT images with higher levels of consolidation, voids, upper lobe involvement, and enlarged lymph nodes were more likely to come from patients with highly infectious forms of PTB.

**Conclusion:**

Our study proves the feasibility of using CT images to identify the infectivity of PTB patients based on the deep learning method.

## Introduction

Tuberculosis (TB) is a chronic infectious disease primarily caused by *Mycobacterium tuberculosis* (Mtb) (1) and remains the leading infectious reason of death worldwide. Since Mtb is primarily transmitted through respiratory droplets [e.g., coughing, sneezing, speaking, singing (2, 3), and even deep exhalations (4)], pulmonary tuberculosis (PTB) is the most common form of TB. However, despite the crucial importance of rapidly identifying the infectivity of PTB patients for the prevention and control of TB, it remains a task to date.

Etiological examinations are commonly employed to determine the infectivity of tuberculosis patients, yet in China, only 37% of PTB patients receive bacteriological evidence (5). Clinical routine sputum acid-fast bacilli smear (we refer to it as “sputum smear” in the following text) is relatively quick but has poor repeatability and a low single detection rate, often necessitating repeat testing for PTB patients. Sputum culture examination takes 2–6 weeks, and polymerase chain reaction (PCR) tests for Mtb may yield false positives and are costly (6). Moreover, traditional methods for assessing the infectivity of PTB patients rely on the quality of sputum samples, which can be influenced by the operator's skill and the patient's condition. Therefore, there is an urgent need for a rapid, reliable, and objective method to determine the infectivity of PTB patients. CT imaging plays an essential role in analyzing and diagnosing PTB patients (7). Research has also shown that imaging findings are often associated with positive sputum smear results in PTB patients (8–10).

Deep learning is a highly versatile tool widely employed for diagnosing and predicting a wide range of diseases. In the field of PTB analysis, Li et al. (11) combined autoencoder and convolutional neural network (CNN) to proposed a new model called AECNN for abnormal classification of TB. Tian et al. (12) proposes a lightweight classification network based on a combination of transformer and CNN for the classification of TB cases from lung CT. Besides, some studies have used deep learning models for identification of drug-resistant and non-drug-resistant Mtb (13), detection of Mtb and *Nontuberculous Mycobacterium* infections (14), and rapid screening of patients with active PTB (15–17). However, few studies focused on the detection of the infectivity of PTB patients, which is crucial for PTB prevention and control.

In this study, we present a PTB infectivity identification model, named TBINet, which utilizes a 2D projection-based CNN to detect individuals with contagious PTB.

## Methods

### Patients and dataset

We retrospectively collected data from patients diagnosed with PTB who were admitted to four hospitals from January 2010 to December 2021. As sputum smear result is associated with the infectivity of PTB (18), we used sputum smear result to assess infectivity. According to the sputum tuberculosis smear interpretation criteria in the WS 288-2017 (19), the sputum smear result was categorized into six grades from low to high: negative (–), weakly positive (±), positive (+), positive (2+), positive (3+), and positive (4+). A negative result (–) indicates the absence of acid-fast bacilli in 50 consecutive microscopic fields. A weakly positive result (±) indicates the presence of 1–9 acid-fast bacilli in 50 microscopic fields. The positive (+) category refers to 10–49 acid-fast bacilli found in 50 microscopic fields. positive (2+) indicates 1–9 acid-fast bacilli found in each microscopic field, and positive (3+) indicates 10–99 acid-fast bacilli found in each microscopic field. The positive (4+) indicates more than 100 acid-fast bacilli found in each microscopic field. The report for positive (2+) should be based on the observation of a minimum of 50 fields, while for positive (3+) and higher positive results, a minimum of 20 fields should be observed.

We defined the negative group as having three or more negative sputum smear results within a month, and no positive results within the next 3 months, and this indicates weak infectivity. The positive group was defined as having at least one-time positive sputum smear results within a month, indicating relatively strong infectivity. We selected the highest number of multiple sputum smear results for each person in the positive group as the grading criterion. The National Health Commission of the People's Republic of China's diagnostic standards for PTB served as the foundation for the diagnosis of PTB (WS 288-2017) (19). After the patients were grouped according to the sputum smear results, chest CT images of the patients in DICOM format were collected and matched. The interval between sputum smear tests and CT image acquisition was <30 days, and patients with unclear PTB diagnoses or poor-quality lung CT images were excluded.

**Figure 1:**
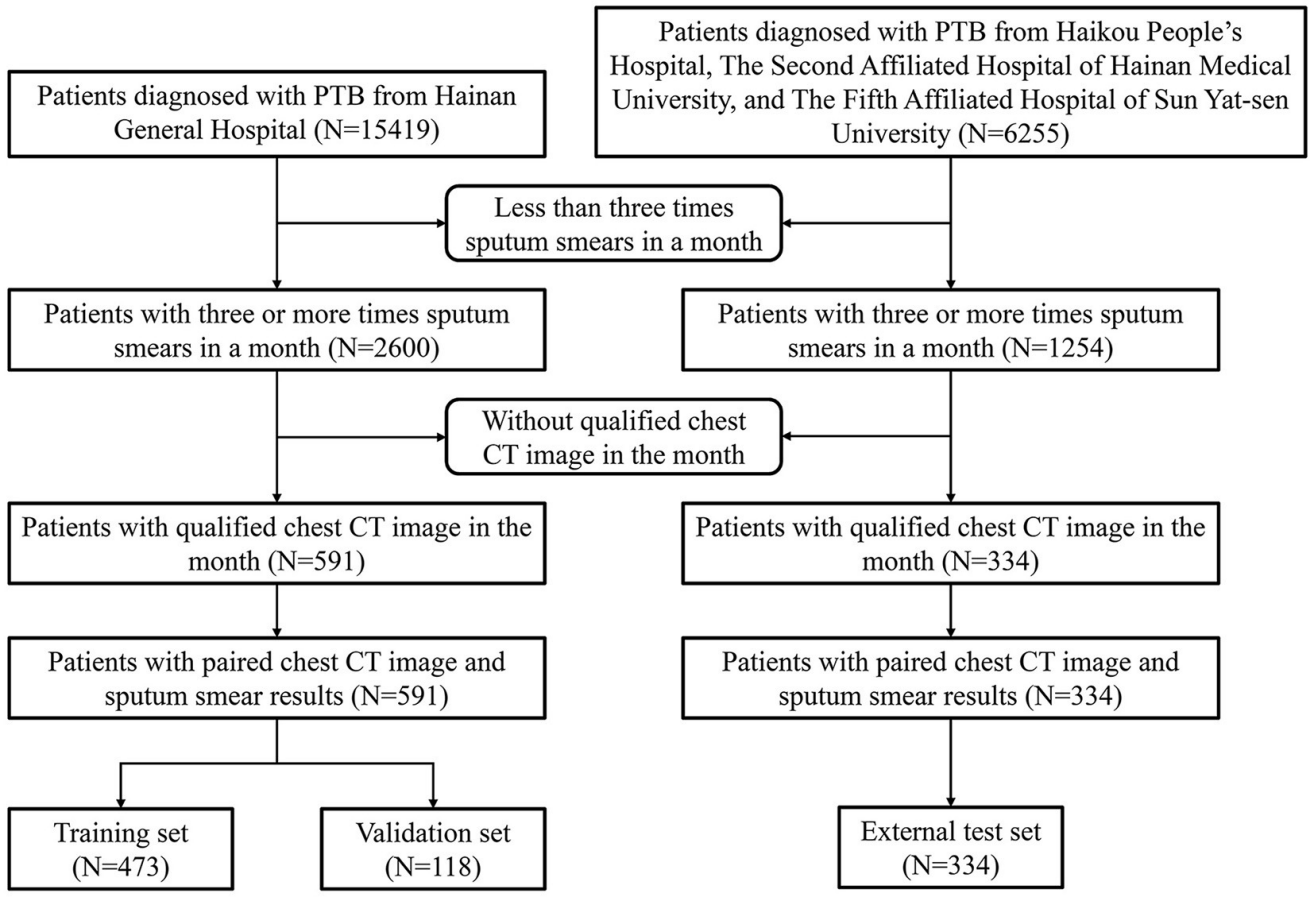
Clinical data screening process and composition.

Figure 1 displays a detailed flowchart of the procedure for gathering data. We included one CT scan image from each patient, totalling 925 CT scan images. Of these, 591 images were split for training, with 118 images set aside as a validation set for model parameter selection. The remaining 334 images were used for testing. Hospital 1 provided the images for the training and validation sets. Hospitals 2, 3, and 4 provided the images for the external test set.

### Data collection

The patients were positioned prone and instructed to inhale and hold their breath as much as possible during the lung field scan. The visual field was adjusted to fit the size of each patient. All patients were scanned using spiral CT scanners following the same protocol. The CT images have an in-plane pixel spacing of 5 mm, an in-plane resolution of 512 × 512, and the number of slices ranges from 47 to 70. All CT data were converted from the original DICOM format to the NIFTI format to ensure data desensitization.

### Lung segmentation

**Figure 2:**
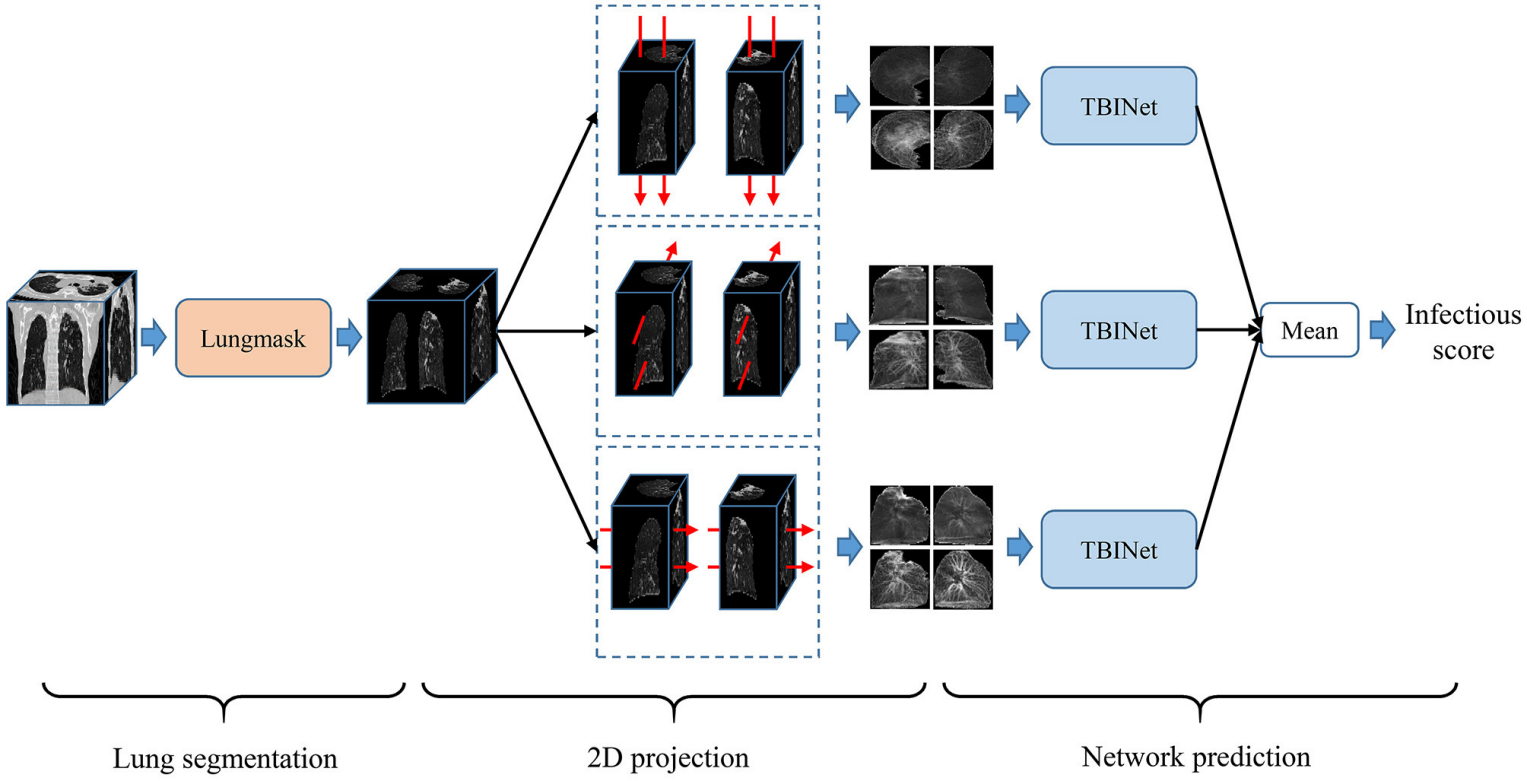
Overview of our proposed infectious PTB infectious scoring method.

Figure 2 shows the overview of our PTB infectious distinguish method. The first step is lung segmentation. Considering that contagious PTB occurs in the lung parenchyma, lung region segmentation can make the model focus on the lung without interference from other areas, thus reducing the difficulty of the analysis. Lungmask (20) is an open-source lung segmentation model based on deep learning, which is used to perform automatic segmentation of lungs on 3D CT images.

### 2D projection

**Figure 3:**
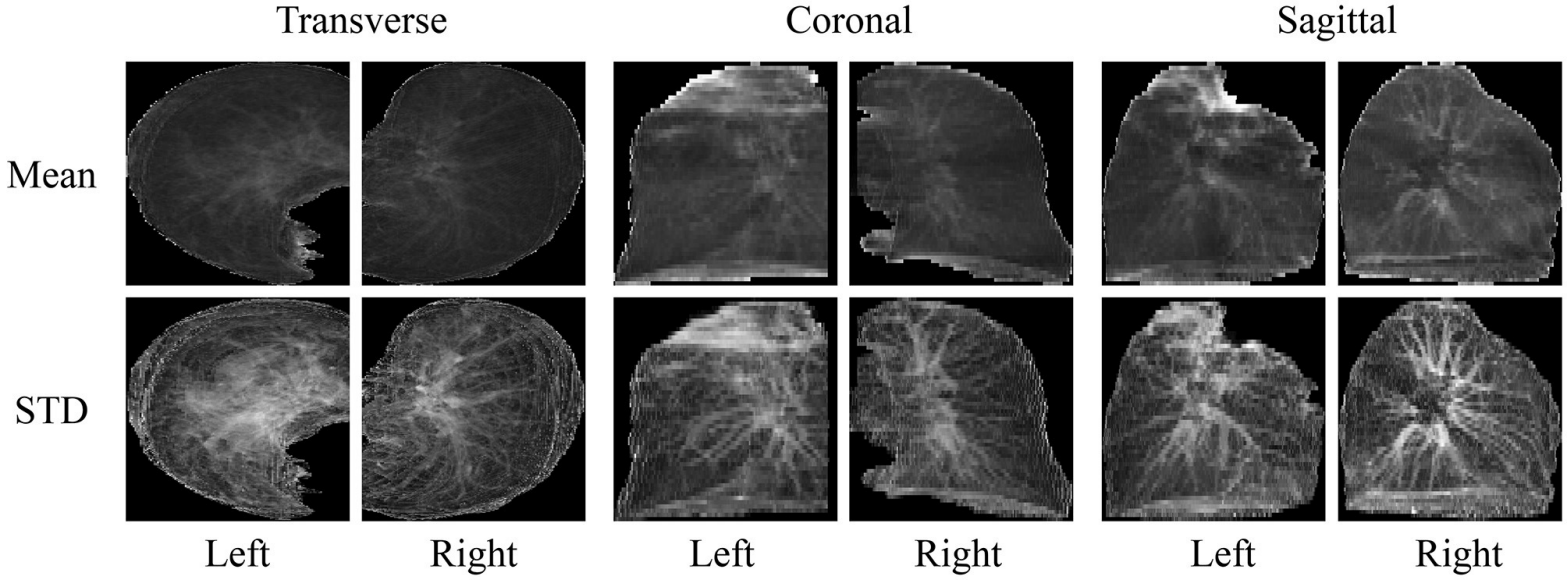
2D projection results generated from 3D masked CT.

Based on the segmented lung mask, a CT image is divided into left and right lung images. Then the left and right lung images are projected from three sides, that is, the mean and standard deviation of pixels in three directions are calculated. Finally, the images of all planes are scaled to a uniform size, as shown in Figure 3. The advantage of projecting 3D images to 2D images is that the 2D CNN can be used to analyze these data. Compared with 3D CNN, 2D CNN is lighter and easier to train. Besides, 2D projection increases the number of samples, because a 3D CT image is converted into three 2D projection samples in different directions, which can alleviate the over-fitting of our model.

### Network construction

**Figure 4:**
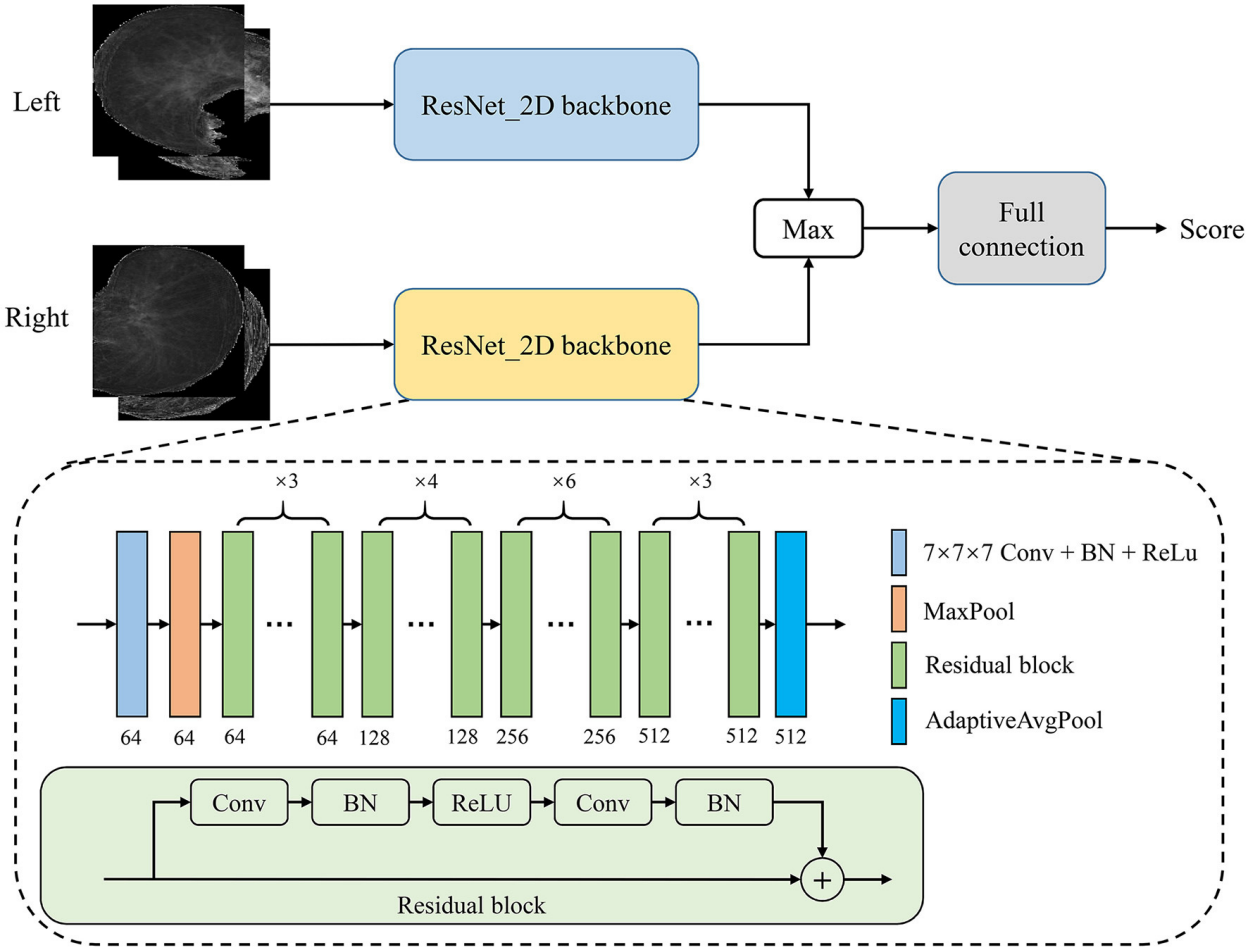
Framework of our proposed TBINet.

In this study, we proposed a PTB infectious convolutional neural network (CNN) which can be called TBINet for rapid identification of contagious people with PTB. As shown in Figure 4, the TBINet contains two ResNet (21) backbones used to extract the features of left and right lung projection images, respectively. The details of the ResNet backbone are also shown in Figure 4, which consists of convolution (Conv) layers, batch normalization (BN) layers, rectified linear units (ReLU), residual blocks, max pool layer, and adaptive avgpool layer. Then the extracted left and right lung features are fused by maximum operation. Finally, the fused features pass through a full connection layer to get the final infectious prediction score. The prediction score is a value between 0 and 1. All the prediction scores can be divided into two groups by setting the cut-off value, with those above the cut-off value being positive and those below being negative.

### Network optimization

A loss function is typically necessary for CNN optimization. For TBINet optimization, we employ a binary cross-entropy loss function, which is specified as follows:


$$\begin{array}{l} L=\frac{1}{N}\underset{i}{\sum }-\left[y_{i}\cdot \log\left(p_{i}\right)+\left(1-y_{i}\right)\cdot \log\left({1-p}_{i}\right)\right] \end{array}$$

Where *N* is the number of samples, *y*_*i*_ is the label of the sample *i*, and *p*_*i*_ is the score of the sample *i* predicted by our TBINet.

The following describes a few implementation specifics. Our model was trained for 300 epochs using the Adam (22) optimizer and a step-decay learning rate. The learning rate was 0.0001 at the beginning. Some straightforward online data augmentation techniques were applied to the training set to reduce overfitting, such as random flipping, rotating, and zooming.

### Model evaluation

The dataset was split into three sets: a training set, which was used to train the TBINet; a validation set, which was used for model parameter selection; and an external test set, which was used to evaluate the generalization ability of our model. We contrast our TBINet with the existing deep learning-based PTB classification methods, including 3D ResNet (14), AECNN (11), and LightCN (12), to demonstrate the advantages of our approach. The area under the receiver-operating-characteristic curve (AUC), accuracy, sensitivity, specificity, precision, and F1 score were calculated for these models to be evaluated and compared in the validation and external test set. The confusion matrices were also computed to display the prediction results of all compared methods.

### Statistical analysis

The age difference was compared using the *t*-test, and gender was evaluated using the Chi-square test. The 95% confidence interval (CI) of the AUC metric was calculated for model evaluation.

SPSS statistical software version 26.0 (IBM Corp., Armonk, NY) and Python software version 3.6.6 (Python Software Foundation, Wilmington, DE, USA) were used for all analyses and model construction. All statistical tests were 2-sided, and *P* < 0.05 were considered to be statistically significant.

## Results

### Patients characteristics

This study included images from 925 in-patients. Both the training and validation sets were obtained from Hospital 1 (Table 1), whereas the external testing dataset was collected from Hospitals 2–4. There were 721 (77.9%) males and 204 (22.1%) females with a mean age of 50.6 ± 17.3 years. The ratio of positive to negative was 1.92, and training, validation, and testing sets were set at 51.1, 12.8, and 36.1% of the full set, respectively. There were no significant differences in the sex ratio between positive and negative groups (*P* = 0.432) and age (*P* = 0.192; see Table 2).

**Table 1:** Characteristics of patients at each hospital.

**Characteristics**	**Number of cases**				
	**Overall**	**Hospital 1**	**Hospital 2**	**Hospital 3**	**Hospital 4**
**Groups**					
Training set	473	473	0	0	0
Validation set	118	118	0	0	0
External test set	334	0	316	6	12
**Sputum smear results**					
Positive group	609	462	147	0	0
Smear ±~+	160	137	23	0	0
Smear 2+	235	137	98	0	0
Smear 3+~4+	214	188	26	0	0
Negative group	316	129	169	6	12
**Gender**					
Male	721	455	255	5	6
Female	204	136	61	1	6
Age, Means ± SDs, years	50.6 ± 17.3	49.6 ± 16.8	52.3 ± 12.0	63.0 ± 12.0	40.6 ± 16.7

Hospital 1: Hainan General Hospital.

Hospital 2: Haikou People's Hospital.

Hospital 3: The Second Affiliated Hospital of Hainan Medical University.

Hospital 4: The Fifth Affiliated Hospital of SunYat-sen University.

**Table 2:** Age and sex comparison between positive and negative groups.

**Characteristics**	**Positive group**	**Negative group**	**Total**	***P*-value**
Male	460	261	721	
Female	149	55	204	0.432
Total	609	316		
Age, Means ± SDs, years	48.4 ± 17.2	51.3 ± 18.3		0.192

### Performance of the TBINet

**Figure 5:**
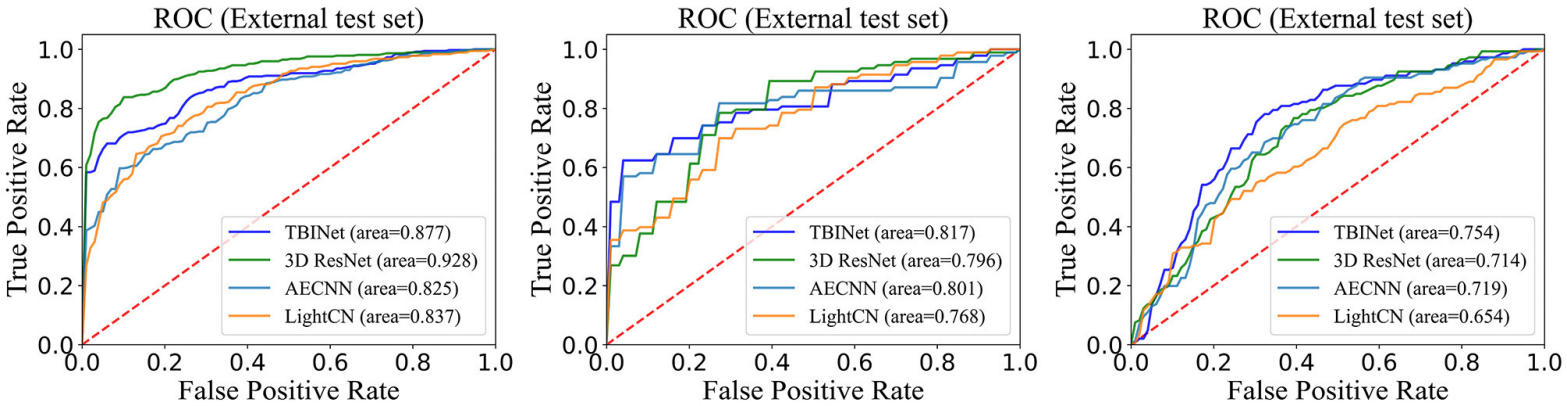
Receiver-operating-characteristic curves on the training, validation, and external test.

**Figure 6:**
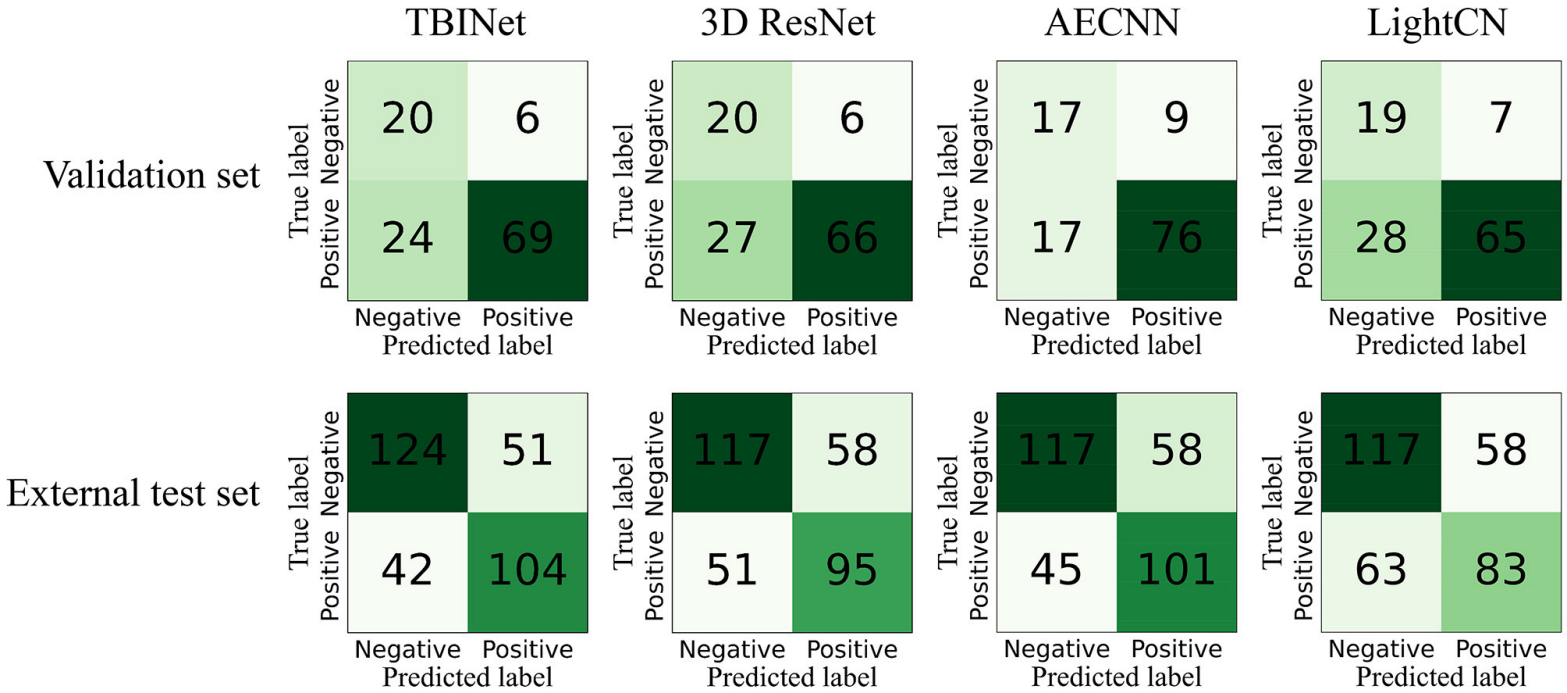
TBINet and 3D ResNet confusion matrices.

Figure 5 shows the receiver-operating-characteristic curves (ROCs) of all compared methods. The 3D ResNet achieves the highest AUC (0.928) on the training set, but a lower AUC on the validation (0.796) and external test set (0.714), which indicates that it has serious over-fitting. Our TBINet achieves the best performance on the validation and external test sets with AUC of 0.817 and 0.754, respectively, which shows the superior generalization ability of our model. The detailed comparisons of AUC, accuracy, sensitivity, specificity, precision, and F1 score on the validation and external test sets are listed in Tables 3, 4, respectively. The results show that the proposed TBINet achieves the best performance with all metrics on the validation and external test set. As shown in Figure 6, the confusion matrices also show the predictions of the TBINet have fewer false positives and false negatives.

**Table 3:** Performance comparison of all methods on the validation set.

**Performance**	**TBINet**	**3D ResNet**	**AECNN**	**LightCN**
AUC	**0.817**	0.796	0.801	0.768
(95% CI)	**(0.730, 0.885)**	(0.680, 0.884)	(0.707, 0.873)	(0.658, 0.857)
Accuracy	**0.747**	0.722	0.747	0.705
Specificity	**0.730**	0.730	0.730	0.692
Sensitivity	**0.741**	0.709	0.741	0.698
Precision	**0.920**	0.916	0.920	0.902
F1 score	**0.821**	0.799	0.821	0.787

The bold values indicate the best results.

**Table 4:** Performance comparison of all methods on the external test set.

**Performance**	**TBINet**	**3D ResNet**	**AECNN**	**LightCN**
AUC	**0.754**	0.714	0.719	0.654
(95% CI)	**(0.697, 0.805)**	(0.657, 0.768)	(0.661, 0.773)	(0.592, 0.711)
Accuracy	**0.710**	0.660	0.679	0.598
Specificity	**0.702**	0.662	0.662	0.588
Sensitivity	**0.712**	0.650	0.691	0.602
Precision	**0.670**	0.620	0.635	0.553
F1 score	**0.691**	0.635	0.662	0.577

The bold values indicate the best results.

### Model explainability

**Figure 7:**
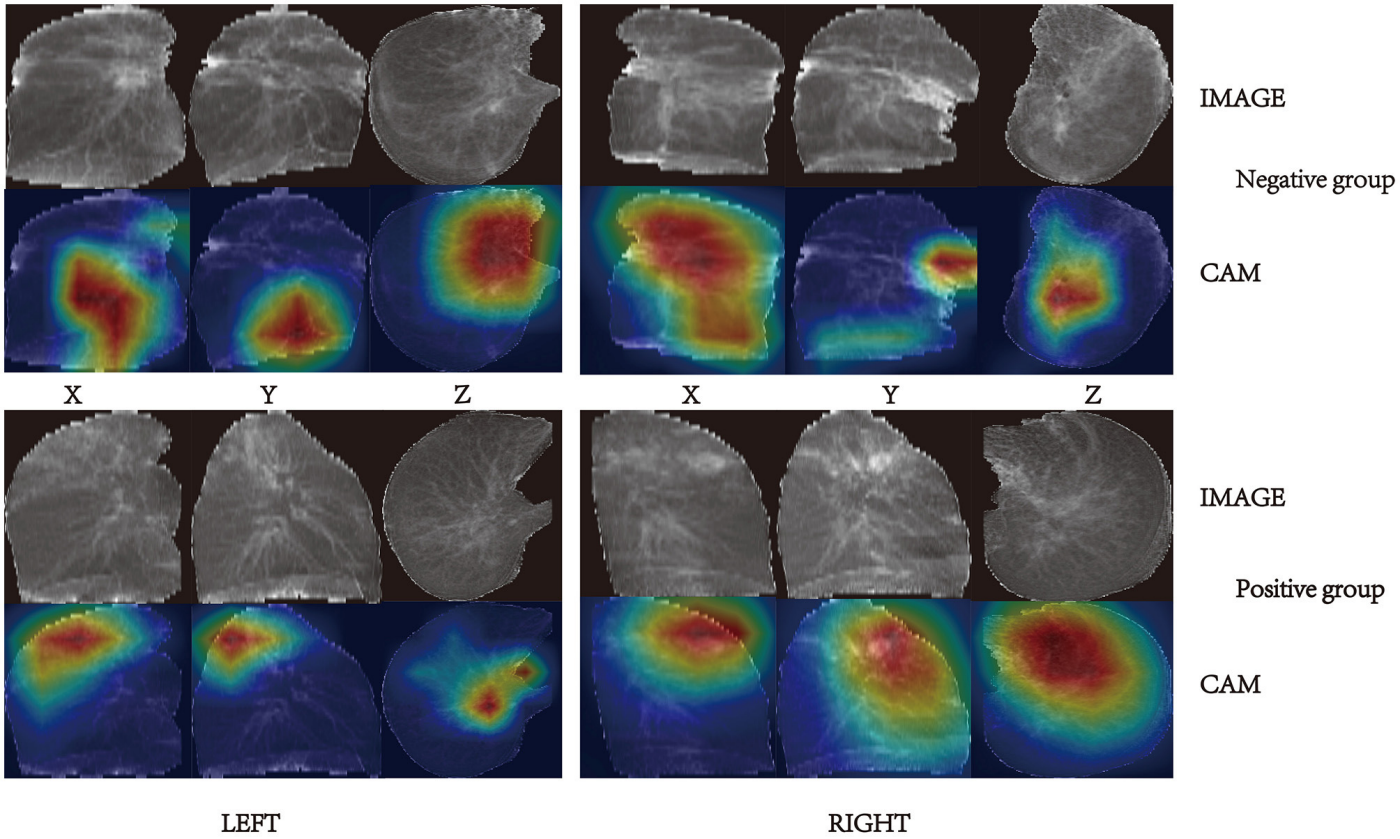
Lesion sites that the models focus on shown by Grad-CAM.

Gradient weighted class activation mapping (Grad-CAM) is a commonly used tool for CNN explainability. It uses gradients of a specific target that flow through the convolutional network to localize and highlight regions of the target in the image. Grad-CAM can reveal the areas of the image that the model relies on to make positive or negative predictions. Figure 7 presents some examples of Grad-CAM in action on our TBINet model.

By observing and analyzing the image areas activated by Grad-CAM, we discovered: in the negative group with weak infectivity, the lesions were characterized by fibrous proliferation foci, bronchiectasis, pleural thickening, stretching, and adhesion, with a few cases exhibiting cavities; in the positive group with strong infectivity, the lesions were primarily characterized by exudate, and some cases exhibited caseous pneumonia, accompanied by cavity formation. These findings are consistent with the pathological features of pulmonary tuberculosis (PTB) (22).

## Discussion

This study aimed to develop a convenient tool for identifying contagious PTB cases, to aid in the prevention and management of tuberculosis. Deep learning methods have recently shown great promise in disease diagnosis and prediction (23–26). Several CNN-based deep learning models have been proposed for TB analysis (17, 27–35). However, few studies have focused on the development of an identification model for PTB infectivity. Furthermore, previous studies have typically relied on etiological specimens to determine the infectivity of PTB patients rather than imaging (36). However, the collection of sputum is often not as convenient and standardized. Therefore, we propose the TBINet, a deep-learning model that utilizes CT images to identify contagious individuals with PTB. Our study demonstrates that the infectivity of PTB patients can be accurately reflected in chest CT images.

To minimize the errors in the label, we defined strict criteria for the inclusion of positive and negative groups. Patients who were included in the negative group need to have three or more records of negative sputum smear results in a month. Previous studies have suggested that having three or more negative smears is sufficient to lift isolation (10). For all enrolled individuals, the time interval between CT scanning and sputum smear testing should not exceed 1 month. To verify the generalization ability, we evaluated our model on an independent external test set composed of data from three hospitals.

Our TBINet uses 2D projection images of CT scans as inputs, reducing the input data's dimensionality. This allows for the use of a lightweight model for image classification tasks, which has better stability and requires fewer graphics processing units (GPUs) (37). Also, this reduces the overfitting of our model compared to 3D ResNet, as shown in Figure 5. While AECNN and LightCN employed 2D neural networks, they analyzed CT images on a single slice, limiting their utilization of 3D spatial information. In contrast, the projection image fed into our TBINet retained 3D information to a degree, and we took into account projections from three directions. During the testing phase, we utilized three TBINet models with shared weights to predict three 2D projection samples from different directions of a CT scan. We then used the mean of their scores as the final result, ensuring that the prediction results of our model were based on comprehensive analysis from multiple angles. According to the 2014 WHO meeting report, a screening test for PTB should have a specificity of over 70% (6, 38, 39). As the output of our model is a score, we suggest setting the cut-off value at 0.64 to maintain a sensitivity of 71.2%.

Previous studies often required each image's region of interest (ROI) for model prediction. However, this process is subjective and requires more workforce (40). In contrast, our model does not require manual ROI, making it convenient, objective, and cheap in the modeling phase. Grad-CAM was used to show the focused areas of our TBINet for explainability. By observing these areas, we found that CT images with more consolidation, voids, upper lobe involvement, and enlarged lymph nodes tended to be in the positive group, which aligns with previous research (9, 10).

CT findings are correlated with the sputum smear results (41). Caseous necrosis and airway lesions form the pathological basis for sputum tuberculosis smear-positive (42). Caseous necrosis, often presenting as consolidations and cavities, can occur when there is an increased number of Mtb (43). Ground-glass opacities and blurred margins are indicative of inflammatory and exudative changes (44), suggesting that the lesions are in the progressive stage, which is related to the host immune response triggered by Mtb replication. Mtb tends to replicate more readily in oxygen-rich areas (43), which can explain why upper lobe involvement is more common in the positive group.

Our study has some limitations. First, our model is unable to determine whether the bacteria discharged by PTB patients were alive or dead, which may require further investigation. Second, although our negative group was labeled based on continuous multiple negative sputum smear results, there were still false negative samples. A multidimensional comparison of Mtb examination results from sputum smears, cultures, and bronchoalveolar lavage fluids is needed to further distinguish PTB infectivity. In the future, our research will focus on seeking a more precise negative group. Third, TBINet cannot explain the process of Mtb replication and release. This may require large cohort studies.

## Conclusion

We developed a deep-learning model called TBINet that can distinguish the infectivity of PTB patients rapidly and cheaply. Experimental results demonstrate that our approach outperforms existing methods. This is a new attempt to distinguish the infectivity of PTB. In resource-constrained regions, it may serve as an auxiliary tool for controlling PTB by aiding in the quick triage and placement of outpatient PTB patients, facilitating secure referrals of PTB patients between various clinical departments, evaluating the condition of PTB patients, and offering personalized assessments of the duration of home isolation for these patients.

## Data availability statement

The raw data supporting the conclusions of this article will be made available by the authors, without undue reservation.

## Ethics statement

The studies involving humans were approved by Hainan General Hospital Ethics Committee (No. 2021-314). The studies were conducted in accordance with the local legislation and institutional requirements. Written informed consent for participation was not required from the participants or the participants' legal guardians/next of kin in accordance with the national legislation and institutional requirements.

## Author contributions

YG: Conceptualization, data curation, formal analysis, funding acquisition, investigation, methodology, project administration, and writing—original draft. YZha: conceptualization, data curation, formal analysis, methodology, software, visualization, and writing—original draft. CH: investigation, formal analysis, and methodology. PH, JF, FL, KL, XF, RL, and JS: Data collection and data analysis. FC: conceptualization, data curation, supervision, and review. WY: conceptualization, resources, methodology, and review. YZho: conceptualization, funding acquisition, resources, supervision, and review. All authors contributed to the article and approved the submitted version.
